# Energetic and behavioral consequences of migration: an empirical evaluation in the context of the full annual cycle

**DOI:** 10.1038/s41598-023-28198-8

**Published:** 2023-01-21

**Authors:** J. Morgan Brown, Willem Bouten, Kees C. J. Camphuysen, Bart A. Nolet, Judy Shamoun-Baranes

**Affiliations:** 1 Institute for Biodiversity and Ecosystem Dynamics, University of Amsterdam, Amsterdam, The Netherlands; 2 Department of Coastal Systems, NIOZ Royal Institute for Sea Research, Texel, The Netherlands; 3 Department of Animal Ecology, Netherlands Institute of Ecology, Wageningen, The Netherlands

**Keywords:** Animal behaviour, Animal migration

## Abstract

Seasonal migrations are used by diverse animal taxa, yet the costs and benefits of migrating have rarely been empirically examined. The aim of this study was to determine how migration influences two ecological currencies, energy expenditure and time allocated towards different behaviors, in a full annual cycle context. We compare these currencies among lesser black-backed gulls that range from short- (< 250 km) to long-distance (> 4500 km) migrants. Daily time-activity budgets were reconstructed from tri-axial acceleration and GPS, which, in conjunction with a bioenergetics model to estimate thermoregulatory costs, enabled us to estimate daily energy expenditure throughout the year. We found that migration strategy had no effect on annual energy expenditure, however, energy expenditure through time deviated more from the annual average as migration distance increased. Patterns in time-activity budgets were similar across strategies, suggesting migration strategy does not limit behavioral adjustments required for other annual cycle stages (breeding, molt, wintering). Variation among individuals using the same strategy was high, suggesting that daily behavioral decisions (e.g. foraging strategy) contribute more towards energy expenditure than an individual’s migration strategy. These findings provide unprecedented new understanding regarding the relative importance of fine versus broad-scale behavioral strategies towards annual energy expenditures.

## Introduction

Seasonal migrations are used by many animal taxa where, by moving between two distant areas, they can exploit seasonal resource peaks, while avoiding deteriorating conditions in the same region during another part of the year^1,2^. Where an individual migrates to spend the nonbreeding season greatly influences both the environmental conditions they experience throughout much of the year (e.g. climate, habitat), as well as the time, energy and risk associated with arriving there, which together we refer to as their ‘migration strategy’. While we have assumptions regarding the costs and benefits of migrating, many of these consequences have yet to be quantified across different migration strategies, particularly how they trade-off across different stages in the annual cycle.

Energy expenditure is perhaps the most apparent cost of migrating, as locomotion is among the most energetically demanding behaviors performed by animals^3–5^. While the process of migrating may be energetically demanding, residency can also lead to elevated energy expenditure for homeothermic animals if they have to increase their resting metabolism to thermoregulate during the winter^6^, or if they have to increase foraging effort due to lower food abundance^7^. This trade-off may result in similar annual energy expenditures (AEE) across a range of migration strategies^8–12^. However, even if annual energy expenditure is similar among strategies, it may be distributed differently throughout the year. As migration distance increases, energy expenditure may shift from being relatively constant throughout the year to more variable, with energy expenditure being concentrated during migration stages, balanced by below average energy expenditure during the winter due to reduced thermoregulatory costs. Higher variability in daily energy expenditure (DEE) throughout the year may pose additional energetic challenges, as animals must be capable of finding, digesting, assimilating, and metabolizing sufficient energy to balance their energy budget. If energy expenditure is concentrated into certain periods, resulting in days with above average DEE, animals may reach limits to time available for foraging^13^, or exceed the limits of their metabolism^14–16^ or heat dissipation^17^, thus increasing risk of mortality^18,19^.

Migrating can also occupy a substantial amount of time which would otherwise be available for other key life history stages. These stages include breeding, periods of growth (e.g. molt) and quiescent periods (e.g. wintering)^20,21^, each of which have their own morphological, physiological or behavioral requirements^22,23^. The reproductive stage is typically the most nutritionally demanding stage of the annual cycle, because individuals must acquire sufficient resources not only for themselves, but also to fuel the maintenance, growth and development of their young^24^. Molt is an energetically demanding growth stage in avian species^25–27^, due to the direct costs of growing new feathers, but also from indirect costs such as impaired body insulation^28^ and reduced flight performance^29^. To compensate for this, birds may reduce flight activity^30^ or move to more productive habitats to complete molt^31^. Finally, animals benefit from having time for a quiescent (‘winter’) stage, which may be important for avoiding energetic bottlenecks when resources are limited^10^, while winter conditions are also important for preparing for the subsequent breeding season^32^. Migrating may have implications for whether animals can adequately adjust their behavior within these stages. For example, non-migratory individuals may not be able to decrease activity levels during winter to rest and prepare for the upcoming breeding season if they must increase foraging effort due to resource scarcity or increased thermoregulatory costs^7^. Migration may also limit time available for these stages, for instance long-distance migrants often return later to breeding areas than short-distance migrants, leaving less time to transition into breeding condition^33^. At the most extreme, migration strategy could favor different ordering of annual cycle events. For example, for long-distance migrants moving to warmer, more productive winter regions, it may be beneficial to delay molt until after migrating, whereas short distant migrants or residents should complete molt in summer before resources become limited in winter^20,22,34^.

To assess the costs and benefits of migrating, currencies of interest should be measured throughout the year and across a gradient of migration distances. Ideally this comparison should be made within a breeding population, to control for differences in ecology, physiology, and morphology among species, as well as different environmental conditions among geographic regions^35^. Lesser black-backed gulls (*Larus fuscus*) are a migratory species which exhibit a range of migration strategies. The Dutch breeding populations contain a mix of short distance migrants wintering in the UK (< 250 km) up to long-distance migrants that travel to West Africa (> 4500 km)^36^. Climate conditions, as well as types of available resources (marine, agricultural and/or urban)^37–40^, should differ substantially within their winter range. Over the past decade, location (GPS) and activity (tri-axial acceleration) of lesser black-backed gulls from three Dutch breeding colonies have been recorded year-round^41,42^, which when combined with a bioenergetics model to estimate thermoregulatory costs, enables us to estimate DEE and activity budgets throughout the year for different migration strategies.

In this study, we aim to quantify the energetic and behavioral implications of migrating in the context of the full annual cycle, using lesser black-backed gulls as a study system. We adopt a comparative approach, dividing gulls into four migration strategies based on their geographical wintering regions: France and UK (short-distance, urban and terrestrial winter resources, cold climate), Iberia (mid-distance, urban, agricultural or marine resources, cold interior or moderate coastal climate), North Africa (mid-distance, urban or marine resources, hot climate), and West Africa (long-distance, marine resources, hot climate). To assess the energetic consequences of migrating, we measure and compare annual energy expenditure (AEE) among these migration strategies, with the expectation that AEE will be equal across strategies due to a trade-off between energy allocated towards activity (i.e., migration) versus resting (i.e., thermoregulatory) costs. Next, we examine how constant or variable DEE is throughout the year, as variable DEE may result in periods of the year where it is more challenging to balance the energy budget. We expect DEE will deviate more from AEE as distance to wintering areas increases, as a result of high energy expenditure during migratory periods due to an increased proportion of time spent in flight and below average DEEs during winter periods due to lower thermoregulatory costs. To assess the behavioral implication of migrating we measured time allocated towards different activities (flapping flight, soaring flight, walking, and resting) throughout the year (i.e., time-activity budgets). Our expectation is that migration strategies will have different patterns in time-allocation throughout the year due to different time constraints and habitat availability resulting from using a given migration strategy.

## Results

**Figure 1:**
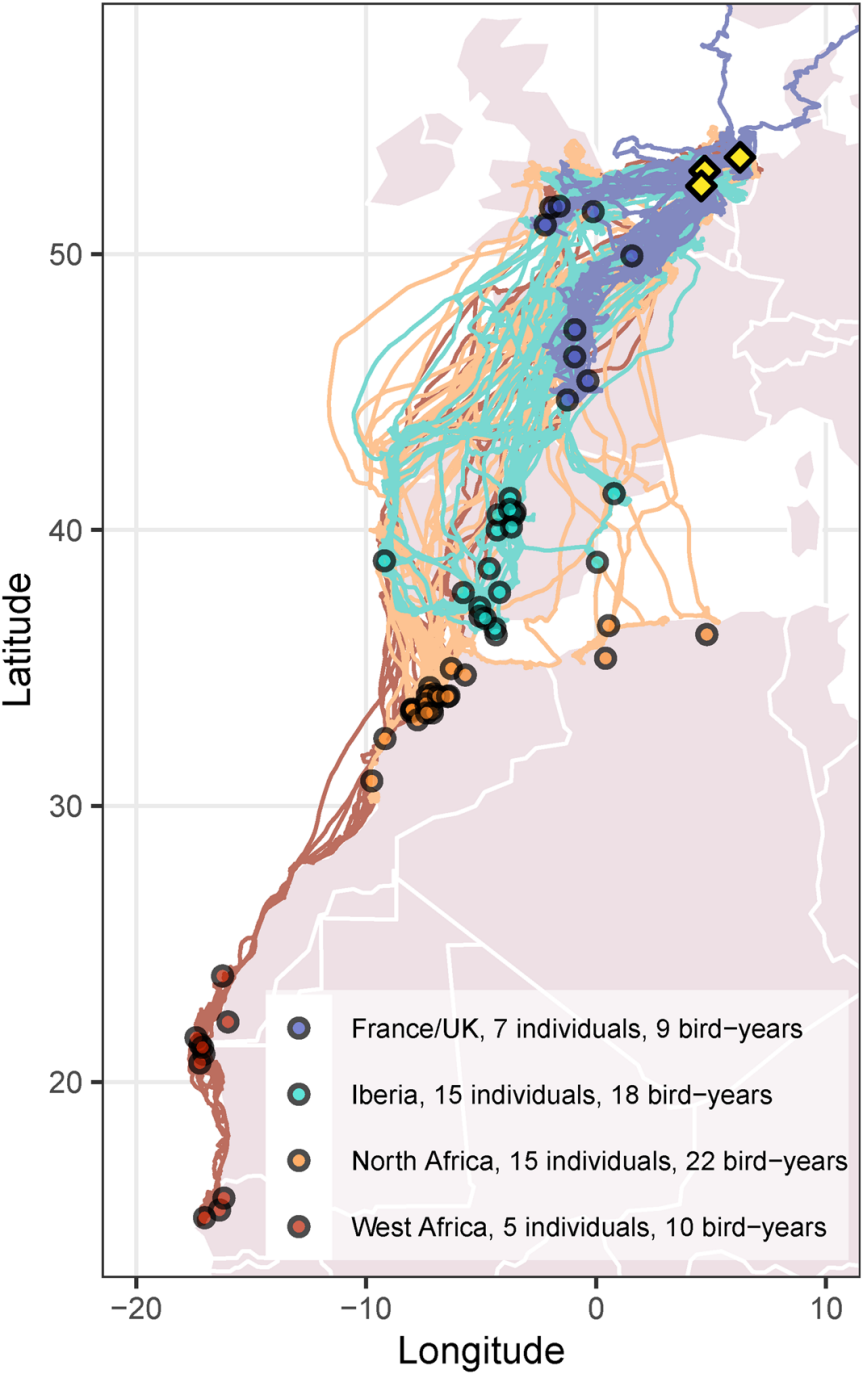
GPS tracks and wintering area centroids (open circles) of bird-years, colored by migration strategy. Breeding colonies are marked with yellow diamonds. Winter areas are jittered to avoid overlap. Map was produced using R packages ggplot^84^ and rworldmap^85^ using Natural Earth data^86^.

A total of 59 bird-years (starting and ending June 1) spanning 5 years (2016–2021) had sufficient full annual cycle coverage. Distance between breeding colonies and winter areas (i.e., migration distance) ranged between 360 to 4583 km with most individuals migrating to either Iberia or North Africa (1350–2872 km, Fig. 1). Several individuals were tracked over multiple years, with 7 individuals migrating to the UK or France (9 bird-years), 15 to Iberia (18 bird-years), 15 to North Africa (22 bird-years) and 5 migrating to West Africa (10 bird-years; Fig. 1). One individual changed migration strategy, contributing one bird-year to the West Africa strategy and one bird-year to Iberia.

### Energetic consequences of migration

**Figure 2:**
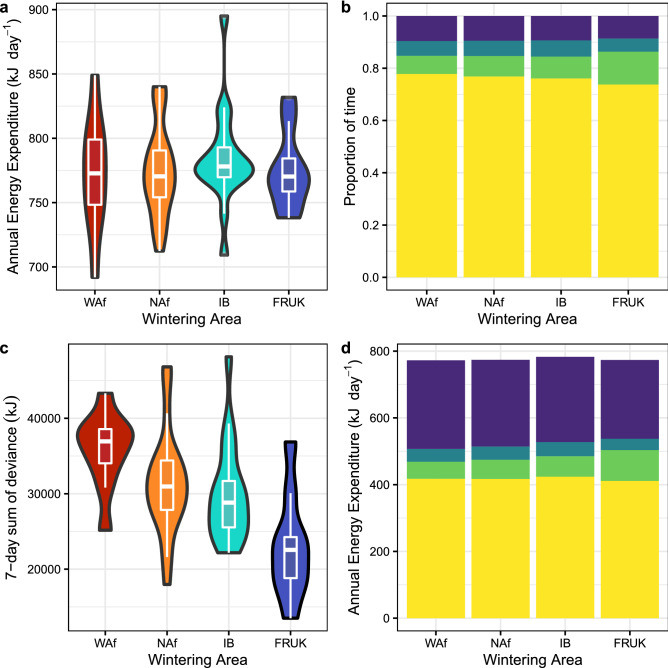
Annual summary of energy expenditure and time-energy budgets by migration strategy. Violin and boxplots show variation in (**a**) annual energy expenditure (AEE) across bird-years, and (**c**) the sum of deviance between AEE and 7-day average DEE. Stacked bar plots show allocation of time (**b**) and energy (**d**) towards different behaviors where purple is proportion flapping, blue is soaring, green is walking and yellow is stationary. WAf = West Africa, 5 individuals, 10 bird-years; NAf = North Africa, 15 individuals, 22 bird-years; IB = Iberia, 15 individuals, 18 bird-years; FRUK = France and UK, 7 individuals, 9 bird-years.

**Figure 3:**
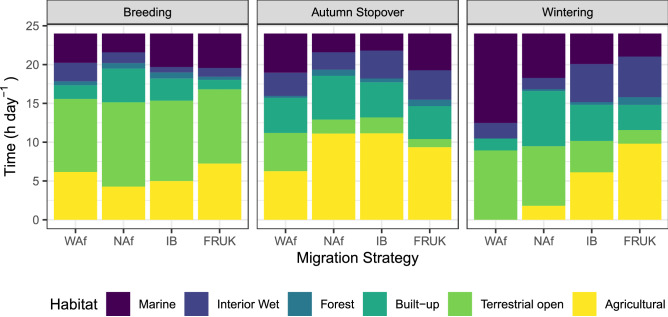
Average time per day spent in each habitat by migration strategy during breeding, autumn stopover, and on wintering area. WAf = West Africa, 5 individuals, 10 bird-years; NAf = North Africa, 15 individuals, 22 bird-years; IB = Iberia, 15 individuals, 18 bird-years; FRUK = France and UK, 7 individuals, 9 bird-years.

AEE did not significantly differ among migration strategies (likelihood ratio test, LRT: df = 3, χ^2^ = 0.70, p = 0.87; Fig. 2a), and linear mixed models (LMMs) predicted it to be 777 kJ day^−1^ (1.96-times the resting metabolic rate, RMR, measured in captivity in a post-absorptive state within their thermoneutral zone)^43^, albeit with large variation among bird-years within a migration strategy (Fig. 2a). For the one individual who changed migration strategy, AEE was 81 kJ day^−1^ higher the year it migrated to West Africa versus Iberia, an increase larger than the median variation in AEE among individuals tracked for multiple years (31 kJ day^−1^, n = 14 individuals), though not the largest change observed (108 kJ day^−1^ in a repeat West African migrant). Habitat use diverged among migration strategies during winter, but not during other annual cycle periods (Fig. 3). Nevertheless, AEE did not vary with the proportion of time an individual spent in any of the main foraging habitats, including marine (LRT: df = 1, χ^2^ = 1.13, p = 0.29), agricultural (LRT: df = 1, χ^2^ = 1.14, p = 0.29) or built-up (LRT: df = 1, χ^2^ = 2.76, p = 0.10) areas.

For all strategies, the majority of the day was spent stationary (Fig. 2b). Time stationary increased slightly, but not significantly, with distance to wintering area (LRT: df = 3, χ^2^ = 5.50, p = 0.14; Fig. 2b). Differences in thermoregulatory costs between winter regions were insufficient to cause significant differences in annual mean resting metabolic rates between strategies (LRT: df = 3, χ^2^ = 4.51, p = 0.21), so average energy allocated towards stationary behavior was likewise not significantly different (LRT: df = 3, χ^2^ = 1.05, p = 0.79; Fig. 2d).

Average time walking per day differed by migration strategy (LRT: df = 3, χ^2^ = 20.93, p < 0.001; Fig. 2b). Individuals using the French/UK strategy spent the most time walking, with LMMs predicting them to walk 2.94 ± 0.27 h day^−1^ (± standard error), almost an hour more than other strategies (Iberia: 2.01 ± 0.19; North Africa: 1.87 ± 0.23; West Africa: 1.74 ± 0.18). Average time spent in either flapping or soaring flight did not differ by migration strategy (Flap LRT: df = 3, χ^2^ = 2.53, p = 0.47, predicted time flapping = 2.22 h day^−1^; Soar LRT: df = 3, χ^2^ = 4.72, p = 0.19, predicted time soaring = 1.40 h day^−1^). Despite only a small portion of time being allocated towards flapping flight, it accounted for a large portion of the daily energy budget (Fig. 2d).

While AEE was unaffected by migration strategy, the sum of deviance between AEE and daily, weekly, and monthly energy expenditure, all increased significantly with migration distance (daily LRT: df = 3, χ^2^ = 15.38, p = 0.002; 7-day LRT: df = 3, χ^2^ = 15.33, p = 0.002; 30-day LRT: df = 3, χ^2^ = 14.92, p = 0.002). This means that, as expected, longer distance strategies have more days, weeks and months during the year with higher and lower than average DEE compared to shorter distance strategies (Fig. 2c).

### Behavioral implications of migration

**Figure 4:**
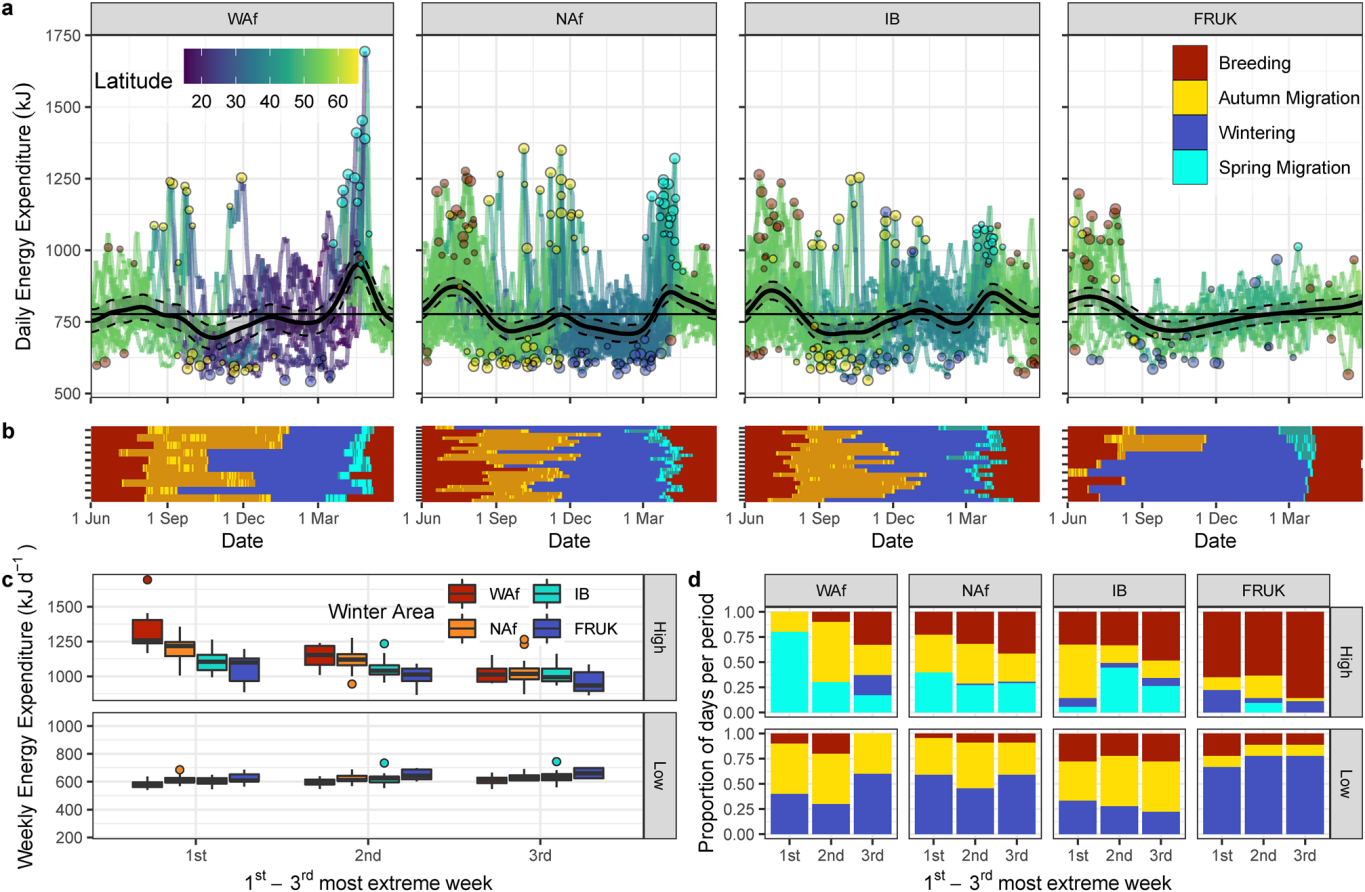
Energy expenditure during the annual cycle. (**a**) Daily energy expenditure (DEE) of lesser black-backed gulls throughout the year by migration strategy (WAf = West Africa, 5 individuals, 10 bird-years; NAf = North Africa, 15 individuals, 22 bird-years; IB = Iberia, 15 individuals, 18 bird-years; FRUK = France and UK, 7 individuals, 9 bird-years). Bold black line shows results of the GAMM model per strategy with 95% point-wise confidence intervals around the fixed effect. Colored lines show 7-day mean DEE per bird-year, colored by latitude. Points mark the three highest and lowest 7-day mean DEEs per bird-year, with point size indicating rank (most extreme being larger), and color indicating the annual cycle stage of the central day of that week. The horizontal line indicates the annual mean DEE across all strategies. (**b**) Timing of annual cycle periods of each bird-year, ordered from longest (top) to shortest migration distance per strategy. Stopover days during autumn and spring are differentiated from migration days by the darker tone. (**c**) Boxplots showing value of three highest and lowest weeks per bird-year, by migration strategy (indicated by colored points in panel (**a**)). (**d**) Stacked bar plot showing distribution of annual cycle stages of the 7 days contributing to the three highest and lowest weeks per strategy.

**Figure 5:**
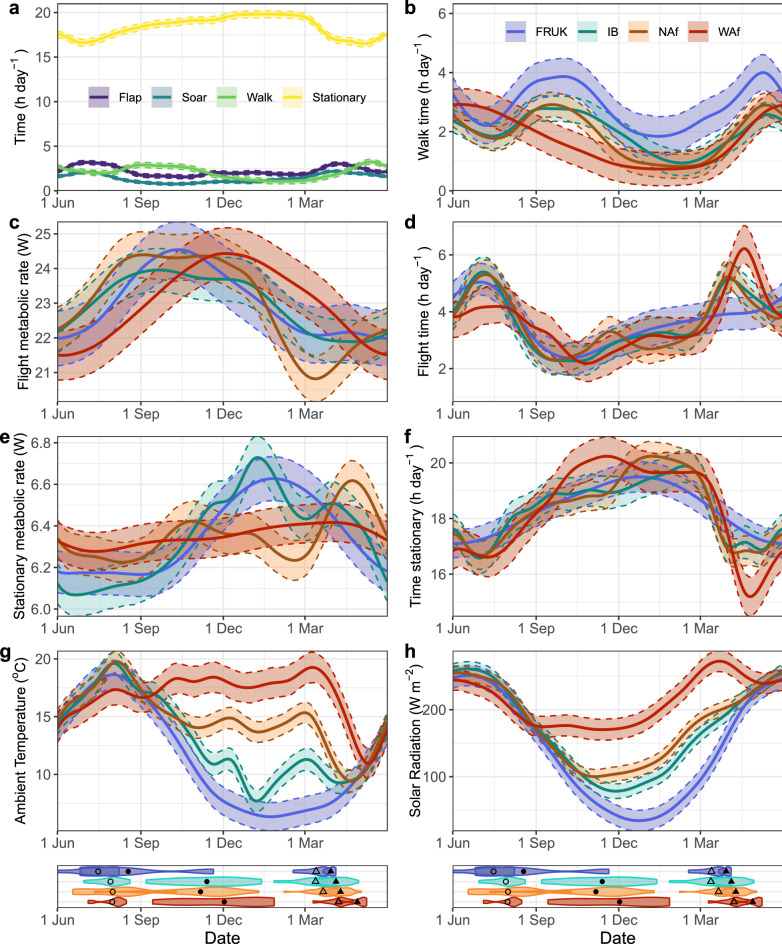
Generalized additive mixed model results of time or energy allocated to different behaviors, and weather conditions throughout the year. (**a**) General pattern across all migration strategies (top model based on AIC), with behavior models distinguished by color. The top model for walking included strategy-specific intercepts, with the French/UK migration strategy walking 0.90–1.16 h day^−1^ more throughout the year compared to the other strategies (not shown). (**b**) Time walking, (**c**) flight metabolic rate. (**d**) Time flying, (**e**) stationary metabolic rate, (**f**) time stationary, (**g**) ambient temperature, and h) solar radiation, by migration strategy (distinguished by colour). Bottom panel shows violin plots of probability density for the start dates of annual cycle stages for each migration strategy, with points indicating the mean per strategy, where open circles are the start of autumn migration, filled circles are start of wintering, open triangle is start of spring migration, and filled triangle is start of breeding. WAf = West Africa, 5 individuals, 10 bird-years; NAf = North Africa, 15 individuals, 22 bird-years; IB = Iberia, 15 individuals, 18 bird-years; FRUK = France and UK, 7 individuals, 9 bird-years. Point-wise 95% confidence intervals around the fixed effects are shown.

DEE (Fig. 4), and time per day spent flapping, soaring, walking and stationary (Fig. 5), fluctuated throughout the year. Migration strategies generally showed similar temporal patterns in DEE and time allocated towards different locomotory modes, with Akaike information criterion (AIC) indicating that a single smoothing function combining all strategies was more parsimonious than strategy-specific smoothing functions for generalized additive mixed models (GAMMs) of all parameters, except for stationary metabolic rate (mean metabolic rate of all stationary points during the day, including thermoregulatory costs) and weather models (Table S1 in supplementary material). The top model for walking included strategy-specific intercepts, with the French/UK migration strategy walking 0.90–1.16 h day^−1^ more throughout the year compared to the other strategies (Table S1, Fig. 5a).

Among individuals using the same migration strategy, both DEE (Fig. 4a) and time-budgets (Fig. S1–S4 in supplementary material) show high variation at a given time. Timing of the main stages in the annual cycle also varied widely between individuals, even when migrating to the same wintering areas (Fig. 4b), diffusing some of the mean patterns within a strategy, particularly the impact of migratory flights. While when considering the entire annual cycle patterns a single smoother was more parsimonious, we present strategy-specific models to identify periods of potential divergence between strategies which may warrant future exploration (Figs. 4, 5b–h).

Daily stationary metabolic rates explained little variation in DEE (correlation, r = 0.26). Instead, DEE was highly correlated with time spent flapping (r = 0.98). Soaring flight, which is an energetically cheaper alternative to flapping flight, accounted for 39% of time in flight. Flight metabolic rate (the mean metabolic rate of all flight points during a day) was highly variable but did show a trend throughout the year, with predicted cost of flight exceeding 22 W in autumn, 2 W more than flight metabolic rate during the rest of the year (Fig. 5c). Variation in daily activity budgets were also correlated with time spent in the three primary foraging habitats used by this species (marine, agricultural and urban/built-up areas). Time in flapping flight increased slightly with time spent in marine habitats (r = 0.30), while time spent walking increased with time in agricultural habitats (r = 0.52) and decreased with time in marine habitats (r = − 0.32). Time stationary or soaring were unrelated to any of the three habitat types (all r < 0.3).

Patterns of DEE generally reflect transitions between annual cycle stages. Peaks in DEE typically coincided with the migration stages or breeding depending on migration strategy, while the magnitude of the peak increased with migration distance (Fig. 4, see also sum of deviance in the previous section). Demonstrating this, the magnitude of average DEE during the three highest and lowest weeks of the year generally became more extreme as distance to wintering area increased (Fig. 4c). West African migrants spent on average 1332 ± 158 kJ day^−1^ (3.36-times RMR) during their highest weekly energy expenditure, which typically occurred during spring migration (Fig. 4d). In comparison, the week with the highest energy expenditure in French/UK individuals typically occurred during the breeding season, and averaged 1059 ± 104 kJ day^−1^ (2.67-times RMR), a similar level reached by the other strategies during that period. Generally, as distance to wintering area decreased, weekly energy expenditure during the migratory periods became more comparable to energy expenditure during the late breeding season (Fig. 4a). Accordingly, the proportion of high energy expenditure weeks occurring during the breeding stage versus during one of the migration periods increased with winter latitude (Fig. 4d). The weeks of the years where DEE was at its lowest generally occurred during autumn stopovers or on wintering areas for all strategies (Fig. 4d). Mean DEE during the lowest weeks of the year also decreased with migration distance, though differences were less extreme (Fig. 4c), with West African migrants having the lowest weekly mean DEE of 581 ± 30 kJ day^−1^, and French/UK migrants having the most moderate mean DEE of 622 ± 40 kJ day^−1^ during their lowest week (1.47 and 1.57-times RMR, respectively).

Patterns in time allocated towards different activities likewise reflected different annual-cycle stages (Fig. 5). Daily time-budgets changed throughout the breeding season. Following arrival to the colony, time in flight initially decreased to moderate levels while time walking briefly increased during the early breeding season (Fig. 5a). As the breeding season progressed, time walking decreased while time in flight increased, with GAMMs predicting of peak at 3.18 h day^−1^ flapping and 2.09 h day^−1^ soaring. West African migrants spent slightly less time in flight during the breeding season compared to the other strategies (Fig. 5d). Around half the time during the breeding season was spent in terrestrial open habitat (i.e., the colony), with individuals from all strategies using a range of foraging habitats (marine, built-up and agricultural, Fig. 3).

Following the breeding season, time spent in flight plateaued at 1.55 h day^−1^ flapping and 0.80 h day^−1^ soaring, while use of walking became more common for a period of approximately 2 months during autumn and early winter, peaking at 2.90 h day^−1^. During autumn stopovers, most individuals were residing in the UK, Belgium, or Northern France (see latitude in Fig. 4a), where use of agricultural and built-up areas increased compared to the breeding season, and time spent in marine areas decreased (Fig. 3). This behavioral pattern is absent in the strategy-specific model for the West African strategy (Fig. 5). Instead, the West African strategy slowly decreased both time walking and in flight throughout the autumn period until activity reached an annual low in early November, when most individuals had already crossed the Sahara. Among-strategy differences in behavior during this period correspond with patterns found in individual bird-years (see ‘Individual-level exploration of autumn time-budgets’ in the supplementary materials), thus may warrant further investigation.

As mentioned previously, peaks in DEE and activity-budgets during migration were not well captured by GAMM models. However, peaks in energy expenditure corresponding with autumn and spring migratory flights are apparent in the 7-day moving mean of individual bird-years in all strategies except French/UK migrants (Fig. 4a). A summary of migration days (Table 1) shows that the length of the migratory period (i.e., time between departure and arrival) was shorter in spring than in autumn by an average of 17 days for French/UK migrants up to 102 days for West African migrants). Seasons did not otherwise differ greatly in number or intensity of migration days within a migration strategy. The number of migration days in a migratory period increased with migration distance, while mean DEE and time in flight during migration days was similar across strategies (Table 1). However, French/UK migrants reached lower maximum DEE and flight times during migration days compared to individuals using other strategies.

**Table 1:** Mean and maximum daily energy expenditure and flight time during migration days by winter area during autumn and spring. Migration days are days with a net displacement greater than 70 km during the migratory period. Length of migratory period is the number days between departure and arrival from the breeding colony or winter area, including stopover days. Reported as mean ± 1 standard deviation.

Winter area	Season	Length mig. period (days)	N. mig. days	Mean DEE (kJ)	Max DEE (kJ)	Mean flight time (h day^−1^)	Max flight time (h day^−1^)
West Africa	Autumn	123 ± 48	18 ± 4	1058 ± 261	1624 ± 214	7.7 ± 3.8	16.6 ± 3.6
	Spring	21 ± 5	14 ± 4	1184 ± 316	1771 ± 276	10.6 ± 3.7	17.3 ± 2.6
North Africa	Autumn	96 ± 46	9 ± 2	1124 ± 316	1752 ± 335	9.9 ± 4.0	17.1 ± 2.8
	Spring	19 ± 9	10 ± 3	1124 ± 117	1653 ± 194	9.3 ± 3.4	15.5 ± 2.1
Iberia	Autumn	106 ± 43	7 ± 2	1140 ± 304	1622 ± 258	9.3 ± 4.1	16.1 ± 2.6
	Spring	23 ± 14	9 ± 3	1085 ± 105	1474 ± 226	8.7 ± 3.2	14.2 ± 2.8
France and UK	Autumn	33 ± 48	5 ± 5	984 ± 219	1211 ± 242	7.8 ± 3.2	11.9 ± 3.0
	Spring	16 ± 16	5 ± 3	1063 ± 172	1222 ± 168	6.6 ± 2.4	10.1 ± 2.1

The winter period was generally the most restful time of the year, with time spent stationary peaking at 19.81 h day^−1^ in mid-January. Time walking decreased compared to autumn stopovers, while flight time increased slightly. Activity budgets remained stable during winter for most strategies, while French and UK migrants steadily increased time in flight as winter progressed. Expectedly, weather conditions differed between wintering areas (Fig. 5g,h). West African migrants never experienced temperatures below 10 °C (their approximate lower critical temperature), while some Iberian and French/UK migrants experienced mean temperatures below 10 °C throughout the winter period. Winter solar radiation and ambient temperature increased with migration distance. As a result, birds overwintering in France, UK and parts of Iberia had higher stationary metabolic rates during winter (Fig. 5e) than birds overwintering further south. The use of different foraging habitats also diverged between strategies (Fig. 3). Use of marine habitats was highest in the West African strategy and decreased in higher latitude winter areas. The French/UK strategy spent half their time in agricultural areas, the use of which decreased as migration distance increased. Use of built-up habitats was highest in the North African strategy, and lowest in West Africa. Despite these differences, all strategies reduced their activity-levels during winter, albeit with French and UK migrants continuing to walk more compared to other strategies.

## Discussion

In this study, we aimed to quantify the energetic costs and behavioral implications of migrating. We found that annual energy expenditure did not differ among migration strategies, and variation among individuals was high within a strategy. Further, the deviance between daily, weekly, and monthly energy expenditure increased with migration distance, demonstrating that individuals migrating further have concentrated periods of high energy expenditure, balanced by below-average energy expenditure, as opposed to being constant throughout the year. Time-budgets also did not differ with migration strategy when comparing the entire annual cycle together, though we briefly discuss some potential differences that may warrant future exploration.

### What are the energetic consequences of migration strategy?

Annual energy expenditure did not differ significantly among migration strategies, in line with our expectation. However, energy allocated towards both active and resting processes did not change with migration strategy, indicating flight costs when migrating did not trade off against lower thermoregulatory costs during winter^44^. Rather, increased flight time during migration as migration distance increased was balanced by slightly lower activity during other annual cycle stages^41^.

Further, instead of clear divisions among wintering areas in annual time and energy allocation, there was high inter-individual variation in annual energy expenditure among individuals migrating to the same wintering areas. Migration strategy is a behavioral decision that acts on broad spatio-temporal scales, influencing seasonal weather and habitat availability. Animal can also respond to their environment on finer scales which are nested within broad-scale behavior strategies^45^. For example, on the finest scale, animals can modify their movement behavior in response to immediate changes in the landscape as they move through it^46^, such as a bird switching from flapping to soaring flight when it encounters rising air^47^. On hourly to daily scales, animals can make decisions regarding where they go to forage, and how they distribute their time between different activities^48^. We speculate that the cumulative effects of these day-to-day decisions, which likely drive the range of variation observed among individuals using the same migration strategy, may be more influential in determining annual energy expenditure than where an individual migrates. Habitat use and foraging behavior of lesser black-backed gulls can be complex and variable even within a winter region^37^, and a more in-depth analysis is required to properly quantify the contribution of foraging strategy towards DEE (and thus inter-individual differences in AEE).

While AEE is balanced across strategies, energy expenditure was more concentrated into periods of several days or weeks as migration distance increased versus being constant throughout the year. Periods of elevated energy expenditure may make obtaining sufficient calories to meet these energy requirements more challenging, and fitness could be negatively impacted if energy expenditure increases above the limit at which the body can assimilate and metabolize energy^13,15,16^ or dissipate heat^17^. The annual cycle stages where gulls experience above average energy requirements were during late breeding and migration, with increasing migration costs for long-distance migrants likely contributing to the increased deviance between DEE and AEE throughout the year. However, even in gulls migrating to West Africa, DEE seldom exceeded 4-times RMR (measured in captivity in a postabsorptive within their thermoneutral zone) during migration days, while weekly peaks were maintained below 3-times RMR. This likely falls within a proposed threshold of 5-time BMR that should allow them to maintain energy balance^49^ suggesting that even during periods of concentrated energy expenditure gulls remain within a sustainable limit. The fact that all migration strategies had similar mean and maximal DEEs on migration days suggests that gulls do limit their daily effort to stay within a sustainable threshold, with long-distance migrants instead choosing to allocate more time (via total number of migration days) to complete their longer migrations. Interestingly, migrants could have dispersed their migration days more evenly throughout the long migration period to achieve more constant energy expenditure. However typically migration days were clustered on either side of long autumn stopovers. Many gulls can be seen undergoing their post-nuptial molt during this period (Sept-Oct, Camphuysen CJ, *unpublished data*). This tactic may enable more separation between migration and molt, emphasizing the importance of separating energetically demanding stages in the annual cycle^23^.

Increased time in flight during the late breeding stage resulted in it being one of the more energetically demanding periods during the annual cycle, though again maximal energetic effort was likely maintained at a sustainable level (well below 4-times RMR). Supporting this, gulls have been observed resting outside of their breeding colony for long periods during the breeding season, emphasizing that they are not devoting all their time towards breeding efforts^50,51^. Elevated DEE as a result of increased breeding effort has been linked to reduced annual survival^18,19^. As a long-lived species with low annual reproductive output, maintaining lower levels of energy expenditure may be a mechanism to optimize long-term survival, and thus future potential breeding opportunities versus investing in current offspring^14^.

When interpreting DEE reported here, it is important to consider that while variation in activity was likely reasonably well accounted for throughout the year in this study, many resting costs are not (e.g., feather molt, and fluctuation in basal metabolic rate due to plastic changes in morphology, physiology, and body mass during different annual cycle stages). Also, our bioenergetic model likely underestimates increased thermoregulatory costs from floating on cold water (due to the higher heat capacity and thermal conductance of water)^52^, as well as resulting from heat stress at elevated atmospheric temperatures^53^. Variation in resting costs throughout the year have been found to significantly influence DEE through time in other species, sometimes more-so than activity^54–57^. In particular, early autumn stopovers appear to be a time of low energy expenditure, however the metabolic costs of feather molt likely do pose an energetic challenge during this stage. In future, measuring of physiological parameters (e.g., heart rate) along-side activity could provide a more detailed picture of variation in energy requirements between annual cycle stages and migration strategies.

### What are the behavioral implications of migration strategy?

Fluctuations in daily activity budgets, when considering the entire annual cycle at once, were very similar among migration strategies. Similarity in behavioral patterns among migration strategies suggests that conditions throughout the entire range currently used by lesser black-backed gulls can appropriately satisfy the behavioral and energetic requirements of the annual cycle stages during which those regions are occupied. Changes in activity-budgets coincided with transitions between different annual cycle stages, demonstrating the need for behavioral flexibility to satisfy changing behavioral and energetic requirements of each stage^58^.

Time-budgets shifted throughout the breeding season. During the incubation period (typically beginning in May), gulls tend to stay closer to the colony and spend more time attending the nest^59^. Chicks begin to hatch in June, when we saw a progressive reduction in time walking with time flapping increasing to the highest level obtained outside of migration, likely resulting from increased foraging effort while provisioning growing chicks^60^. Increasing energy expenditure as the stages of breeding progress from incubation through chick care is commonly found in energetic studies of seabirds^61^. West African migrants seemed to have slightly lower flight time compared to other strategies, though our sample size is low. Combined with the fact that this strategy returns later to the colony^41^, we speculate that West African migrants may be capital breeders, benefitting from a more marine winter diet, whereas the other strategies may be income breeders, investing more time into foraging throughout the breeding period. Alternatively, late returning West African migrants may forgo breeding in some years^62^, reducing the average activity levels of this strategy during the breeding period. (but not individual activity levels if actively breeding).

During early autumn, time in flight dropped to an annual low, accompanied by a marked increase in time spent walking. This behavioral change is consistent with reduced activity observed during the non-breeding season of other seabird species and generally attributed to molt^30,63^. Individuals may be reducing energy invested in activity, especially flight, to manage increased resting energy expenditures resulting from molting. During this period, most individuals remained at higher latitudes for several months after departing their breeding colonies, residing in the UK, Belgium or Northern France, where there are ample agricultural and anthropogenic foraging areas^38^. This may facilitate reducing time spent in flight, resulting in overall lower DEE during this period (before accounting for metabolic costs of feather molt).

Longer-distance migrants, particularly the West African strategy, were less likely to demonstrate behavioral accommodations suggestive of molt than individuals migrating to nearer wintering areas. Long-distance migrants spent more days migrating, reducing time available for other annual cycle stages. This could result in increased temporal overlap between stages^23^. A greater portion of individuals migrating to West Africa showed a molt-like behavioral pattern during the breeding season, which may indicate they overlap molt with breeding. Alternatively, individuals migrating to West Africa may delay or suspend molt until reaching wintering areas, where they likewise are able to substantially reduce both flight and walking activity. This has previously been suggested for colonies in the UK, where gulls were found to have a bimodal pattern in onset of molt, hypothesized to be associated with whether individuals were resident (or short-distance migrants), versus migratory^64,65^.

Despite residing in wintering areas with different foraging habitats, individuals from all migration strategies reduced activity during winter, with a remarkably high proportion of the day spent stationary. While activity and energy expenditure did not differ greatly between wintering areas, environmental conditions do. Weather experienced within the northern parts of winter range increased stationary metabolic rate, but not to a degree that it had a strong influence on DEE. Unfortunately, we are unable to account for differences in resource abundance or energy intake rates between wintering areas and resource types in this system, which may influence winter survival. Variation in nutritional quality of resources during the wintering periods could also potentially lead to carry over effects on reproductive success^66^. A comparative analysis of survival and reproductive outputs may illuminate long-term fitness consequences of using different wintering areas^9,12,67,68^.

Overall, patterns of energy expenditure and time-activity budgets throughout the year were remarkably similar between migrations strategies, suggesting that among all strategies, birds adjust behavior to accommodate the main stages in their annual cycle while maintaining energy expenditure within a sustainable limit. Large inter-individual variation among individuals using the same migration strategy may indicate that the cumulative impacts of fine-scale behavioral decisions may be just as, or more, influential at determining annual energy expenditure than migration strategy itself. This provides unprecedented new insights into the costs and benefits of migration with regards to two key ecological currencies, time and energy allocation. Such costs are often not evaluated throughout the year, and by doing so we are able to assess of how costs experienced during migration relate to and trade off with other annual stages over a range of migration strategies. Our findings demonstrate the importance of, and the need for more, full annual cycle empirical studies on the consequences of migration to advance our understanding of the drivers of migratory life-histories.

## Methods

### Capture and tagging

Breeding adults were tracked from three colonies in the Netherlands as a part of long-term monitoring studies (IJmuiden 52°27′54 N 4°34′34E: 35 individuals between 2019–2021; Texel 53°00′33N 4°43′10E: 104 individuals between 2008–2021; and Schiermonnikoog 53°30′00N 6°15′34E: 29 individuals between 2017–2018). Individuals were captured in a walk-in trap set over a nest during the incubation stage and solar-powered GPS and acceleration trackers (UvA-BiTS)^69^ were attached using a Teflon wing harness^70^. Combined mass of tracker and harness (13.5–15 g) were less than 3% of body mass. GPS fixes followed by 1 s of tri-axial acceleration at 20 Hz were taken year-round at an interval between 2.5–60 min, depending on location, time of year, battery level and available memory. Measurements are stored on the logger and transmitted to a base station in the colony during the breeding season. All capture, handling and tagging procedures were licensed by the Centrale Commissie Dierproeven (AVD8020020174225) and were approved by the animal welfare committee (Instantie voor Dierenwelzijn) of the Netherlands royal institute for sea research (NIOZ), following the Dutch Animal Welfare Act Articles 9, 10 and 11 of the animal experiment documents. All methods were performed in accordance with the relevant guidelines and regulations and are reported in accordance with ARRIVE guidelines.

### Data processing

‘Bird-years’ (starting and ending on June 1) that had GPS and acceleration data for at least 75% of the year were selected from the complete dataset for processing. A behavior was assigned to each acceleration measurement using a random forest classifier previously developed for lesser black-backed gulls^71^. Following methods in^43^, behaviors identified by the classifier were combined into four behavior modes: flapping, soaring, walking and stationary (sitting, standing or floating on water).

Data were subsampled to an interval between 20–60 min, based on the lowest sampling frequency on that given day. To assess whether sample frequency biased our results, we used a high-resolution acceleration dataset from the 2019 breeding season to estimate daily energy expenditure (DEE, see below) for data measured at a 2.5-min interval (highest resolution available), a 20-min interval (most common annual sampling interval) and a 60 min interval (lowest resolution in this study; n = 207 days from 5 individuals). Precision of DEE decreased with sample interval, with the median magnitude of error being 4.7% of DEE (± standard deviation) at the 20-min interval and 8.6% of DEE at the 60-min interval. However, error was not biased so low resolution data neither systematically over- or under-estimated DEE, demonstrated by the linear relationship between DEE estimated from low- versus high-resolution data falling along the line of equality (Fig. S5 in supplementary material).

Net displacement over each day was calculated as the great circle distance between GPS fixes closest to midnight to distinguish between ‘relocation’ versus ‘stationary’ days. There was no clear bimodal distribution in net distance travelled per day, but there was a drop in the frequency of days with net displacements exceeding 70 km, which was used as a threshold to separate relocation and stationary days.

Data gaps commonly occur at high latitudes during autumn and winter when tracker batteries are unable to charge due to short daylight hours and poor weather. Discarding all bird-years with a gap, or periods of years containing a gap, would bias our thermoregulatory estimates towards fair-weather years and regions. Data exploration suggested that gulls tend to allocate time similarly between activities within a stationary period (consecutive stationary days), coinciding with extremely high repeatability in space used observed within wintering areas across all migration strategies^42^. Therefore, we filled gaps with simulated time-activity budgets by randomly sampling GPS-acceleration fixes within the same stationary period and at ± 1 h of the missing measurement. As assessing the contribution of potentially elevated energy expenditures during migration is central to our research question, any bird-year with a bird that relocated during a data gap was discarded. An average of 3.1% of data was simulated for West African migrants, 6.6% for North Africa, 6.9% for Iberia, and 10.8% for France and the UK. The longest period of simulated data was 80 days during the winter period of an individual in the UK, and which accounted for 36.5% of the days during its winter period. Energy expenditure during filled gaps was estimated using the acceleration of the sampled fix, and thermoregulatory costs were calculated using the weather experienced at the sampled location using the date and time of the missing fix (see “Estimating energy expenditure”). Simulated time and energy budgets during stationary gaps were included in analyses of annual summaries, DEE, and stationary metabolic rate, to account for weather (and thermoregulatory costs) during gaps, as well as ensuring the proportion of time spent in each stage is properly reflected (for annual summaries). Simulated time-budgets were excluded from analyses of time allocated per activity.

Habitat classifications were extracted from Copernicus Global Land Service (updated annually between 2016–2019, 100 m resolution)^72^ for each GPS fix at the closest available year. We reduced the number of classifications to six: marine, built-up (i.e. urban), agricultural (croplands, includes most landfill sites), terrestrial open (herbaceous vegetation, bare/sparse vegetation, shrubland), inland wet (permanent inland water and herbaceous wetland), and forest (all forest classification types). Misclassified habitat within the breeding colonies were corrected to terrestrial open habitat.

### Estimating energy expenditure

Metabolic rates for each measurement were estimated using two approaches: the first was from acceleration data, as an estimate of activity costs, and the second is based on thermoregulatory costs, using a bioenergetics model. To estimate metabolic rate from activity, we calculated the vectoral sum of the dynamic body acceleration measured on three-axes (DBA)^73^. First, we removed the baseline acceleration from each axis by subtracting the mean acceleration across the recording chunk. DBA was then calculated as $DBA= \sqrt{{x}^{2}+{y}^{2}+{z}^{2}}$, where *x*, *y*, and *z* are remaining dynamic acceleration in the surge, sway and heave directions. Activity costs were based on energy estimates reported in^43^ from five lesser black back gulls during the breeding season. For non-floating stationary behavior, we converted DBA to metabolic rate using a calibration equation derived for DBA, where metabolic rate (W) = 4.80 + 49.80DBA. Significant behavior-specific calibrations could not be derived for flapping, soaring, walking, and floating, so average costs for each behavior were used, with flapping flight = 32 W, soaring flight = 7.9 W, walking = 8.5 W and floating = 6.6 W.

The metabolic rate required to maintain body temperature was estimated using a model based on heat exchange theory developed for *Calidris* sandpipers^74^, and subsequently adapted for waterfowl (*Anseriformes*)^75^. The model estimates thermal metabolic rate based on ambient air temperature at 2 m (*T*_*a*_, °C), wind speed at 10 m (*u*, m s^−1^) and surface solar radiation (*R*_*g*_, W m^−2^), which were extracted for each GPS fix at the nearest hour and 0.25° latitude-longitudinal grid from the ERA5 dataset^76^. The model was followed as reported in^75^, modifying the species specific constants for gulls (see ‘Constants used in heat-exchange model’ in supplementary material). We did not account for potential increased thermoregulatory costs while floating, which can result from water having higher thermal conductance and greater specific heat capacity than air^52,77–79^. We found gulls did not incur extra thermoregulatory costs while floating in water around 12 °C^43^, though this may lead to underestimation of thermoregulatory costs while floating in colder water. We also did not account for elevated metabolic rates resulting from heat stress^53^, which may underestimate resting metabolic rates in more tropical wintering areas. Finally, we did not account for variation in other resting costs (e.g., feather molt, digestion), and we assumed these costs will be similar across individuals, regardless of migration strategy.

We assumed thermoregulatory costs are compensated by activity, so the highest of these two estimates was selected per fix and averaged over the day to estimate DEE. Daily time-budgets were estimated based on the proportion of fixes per day classified as a given behavior. Mean annual energy expenditure (AEE) was calculated by averaging DEE throughout the year. Flight metabolic rate was the mean metabolic rate of all flight fixes during a day (in W). Stationary metabolic rate was the mean metabolic rate of all stationary fixes during a day (including estimated thermoregulatory costs when they exceeded activity costs, in W).

### Statistical analysis

We partitioned the annual cycle into four stages: breeding, autumn migration, wintering, and spring migration based on GPS locations, following methods in^42^. Breeding was considered the stage when individuals had an association with the breeding colony. The last detection within 10 km of the colony was used for the transition from breeding to autumn migration, and the first detection within 10 km of the colony the following year was the transition from spring migration to breeding. Wintering areas for each bird year were identified from the 95% kernel density estimates of GPS locations taken between colony departure and arrival^42^. This approach identifies core-areas of several hundred km in diameter where gulls spend a substantial amount of time. When multiple core areas were identified, the most southerly is assumed to be the wintering area. The winter period was defined between the first and last stationary day within the wintering area. Migration stages were defined as the periods in the annual cycle between breeding and wintering. Relocation days within the migratory periods are referred to as migration days and stationary days as stopover days.

Bird-years were separated into four migration strategies based on the location of their wintering area: Birds wintering in Africa south of 25° latitude (“West African”), birds wintering in Africa north of 25° latitude (“North African), birds wintering in Spain or Portugal (“Iberian”) and those wintering in France and UK.

Linear mixed models (LMM) with migration strategy as a fixed factor were used to examine whether migration strategy influences AEE and annual mean time and energy allocated to flapping, soaring, walking and stationary behavior per day (total of nine models). Individual was included as a random intercept and we used a gaussian probability distribution. Models were estimated using r package lme4^80^. Likelihood ratio tests (LRT) were performed against a null model with no fixed effects to test whether migration strategy explained a significant amount of variation in the models.

To examine whether distribution of DEE becomes more concentrated within certain periods versus constant as migration distances increase, the deviance between DEE and AEE was summed for each bird-year. To take the temporal sequence of DEE into account, the deviance was also calculated using a 7-day and 30-day moving mean of DEE, to examine whether energy expenditure during periods of a week or month deviated more from AEE as migration distance increased (i.e. as opposed to above average DEEs being balanced by below-average DEEs during a given time period). Sum of deviance per bird-year was modelled in response to migration strategy (fixed factor) using LMMs with individual included as a random intercept and a gaussian probability distribution, and significance of migration strategy was assessed by comparing to a null model using a LRT.

Patterns of DEE, daily time spent flapping, soaring, walking or stationary, stationary and flight metabolic rate, and experienced temperature and solar radiation, were modelled as a function of calendar date using generalized additive mixed models (GAMM), including individual as a random intercept and assuming a gaussian probability distribution, using r-package mgcv^81^. To control for temporal autocorrelation, an auto-regressive moving average (ARMA) correlation structure with one auto-regressive parameter and one moving average parameter was included in all additive models. Autocorrelation structure was selected using Akaike’s Information Criterion (AIC) comparing all ARMA correlation structures up to a maximum of two parameters and validated by plotting the autocorrelation function of normalized residuals^82^. Models were estimated containing either a smoothing function per migration strategy, a single smoothing function combining all migration strategies, or a null model with no smoothing function with time, as well as with and without migration strategy as a fixed factor and compared using AIC.

To relate periods of extreme high or low energy expenditure to stages in the annual cycle, the annual cycle stages of the weeks with the three highest and lowest average DEEs were reported per bird-year. The extreme weeks were determined using a 7-day moving average, and the three highest or lowest weeks did not overlap in time (so no day can contribute towards multiple high or low weeks), with extreme weeks being determined consecutively from most to least extreme. All analyses and figures were generated using R version 4.0.2.

## Supplementary Information

Supplementary Information.

## Data Availability

The datasets and workflow used to produce results are archived on Figshare (10.21942/uva.21583926.v1)^83^.
