# Supplementary material for: Energetic and behavioral consequences of migration: an empirical evaluation in the context of the full annual cycle
Source: Sci Rep. 2023 Jan 21;13:1210. doi: 10.1038/s41598-023-28198-8 (PMC9867707; doi:10.1038/s41598-023-28198-8)
Supplement: Supplementary file 1 — Supplementary Information. [file 41598_2023_28198_MOESM1_ESM.pdf]

Supplementary Materials for

Energetic and behavioral consequences of migration: A full annual cycle comparison in a gull species with diverse migration strategies

J. Morgan Brown, Willem Bouten, Kees C. J. Camphuysen, Bart A. Nolet, Judy Shamoun-Baranes

*Corresponding author. Email: jmbrown@wcs.org

This PDF file includes:

Supplementary Text
Figs. S1 to S5
Tables S1 to S2

Supplementary Text

Methods

Constants used in heat-exchange model

We updated the following constants used in Baveco et al 2011 to adapt our model to lesser black-backed gulls (following notation used in Baveco et al 2011). For body temperate T_b we used a value of 40.4∘C, which was measured using implantable loggers in free-range lesser black-backed gulls (Brown JM, unpublished data). Body radius *r* was calculated from body mass at capture using an empirical relationship (Birkbeback, 1966 reported in Baveco et al 2011). Height of center of mass h_b was 0.2 m. Our plumage resistance r_p is based on the thermal conductance reported for herring gulls *L. argentatus* (0.0385 mL O_2 g^{-1} h^{-1} $°C^{-1}$, reported in Ellis and Gabrielsen 2002, based on measurements in Lustick et al 1978). We convert thermal conductance into Watts assuming 19.8 kJ L^{-1} O_2, based on a respiratory quotient of 0.71 (Bartholomew 1982, Ellis and Gabrielsen 2002), and express it in terms of surface area by multiplying by the mass of the bird and dividing by surface area (C, in W m^{-2} $°C^{-1}$). Plumage resistance (s m^{-1}), r_p was then calculated as $r_p = \rho \cdot cp \cdot C^{-1}$, where ρ (g m^{-3}) is the density of dry air at air temperature, T_a: $\rho = 1292 - (5 \cdot T_a) + (0.01567 \cdot T_a^2)$ (reported in Baveco et al. 2011, based on Robinson et al. 1976), and cp is the specific heat of air (1.010 J g^{-1} $°C^{-1}$). Because gulls stand or perch in open habitats, we assume a vegetation height, h_v, of 0 m and a surface roughness z_m of 0.0005 m, the roughness value reported for sandy dunes and intertidal areas in (De Jong et al. 1999).

Results

Individual-level exploration of autumn time-budgets

The general trend during the autumn period shows a period of elevated walking with reduced time in flight. However, strategy-specific model results for the West African strategy suggest they do not follow this pattern, instead maintaining higher levels of walking during breeding, with both walking and time in flight decreasing throughout the autumn season. Individual bird-years were subsequently explored for this behavioural pattern, using the criterion of a minimum 30-day period where average time walking exceeded 2.5 hours while average time in flight was below 2.5 hours (Fig S1-3). In accordance with the strategy-specific model, only two out of ten bird-years in the West African strategy demonstrated this behavioural pattern during autumn or winter, compared to 59% in North African migrants, 67% of Iberian migrants all nine bird-years in French/UK migrants (Table S2). Instead, three of ten West African bird-years demonstrated this pattern during the breeding season (compared to < 10% in other strategies), while half of the West African bird-years never demonstrated this pattern (compared to 32% of North African migrants, 28% of Iberian migrants, and none of the French/UK migrants).

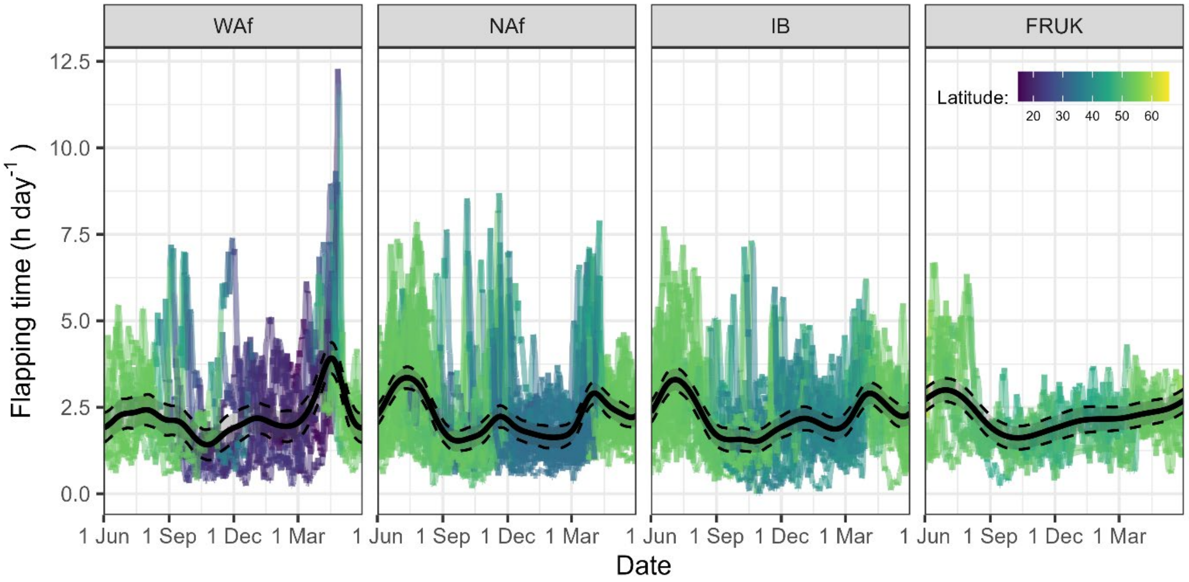

Fig. S1. Daily time in flapping flight by migration strategy (WAf = West Africa, NAf = North Africa, IB = Iberia, FRUK = France and UK). Bold black line shows GAMM prediction per strategy with approximate 95% confidence intervals. Coloured lines show 7-day mean daily energy expenditure per bird-year, coloured by latitude.

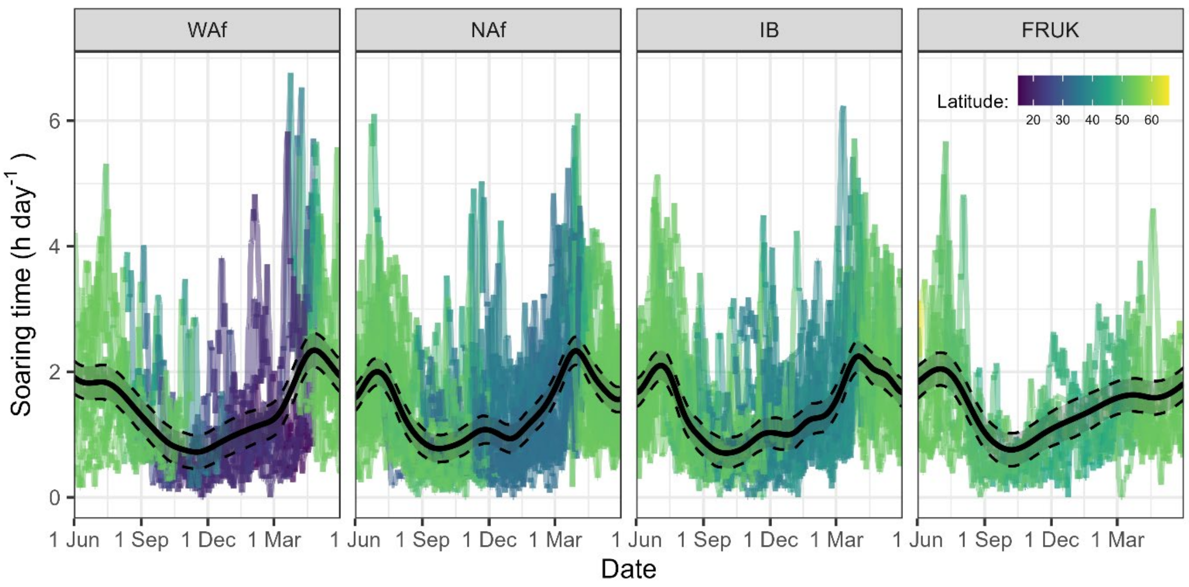

Fig. S2. Daily time spent soaring by migration strategy (WAf = West Africa, NAf = North Africa, IB = Iberia, FRUK = France and UK). Bold black line shows GAMM prediction per strategy with approximate 95% confidence intervals. Coloured lines show 7-day mean daily energy expenditure per bird-year, coloured by latitude.

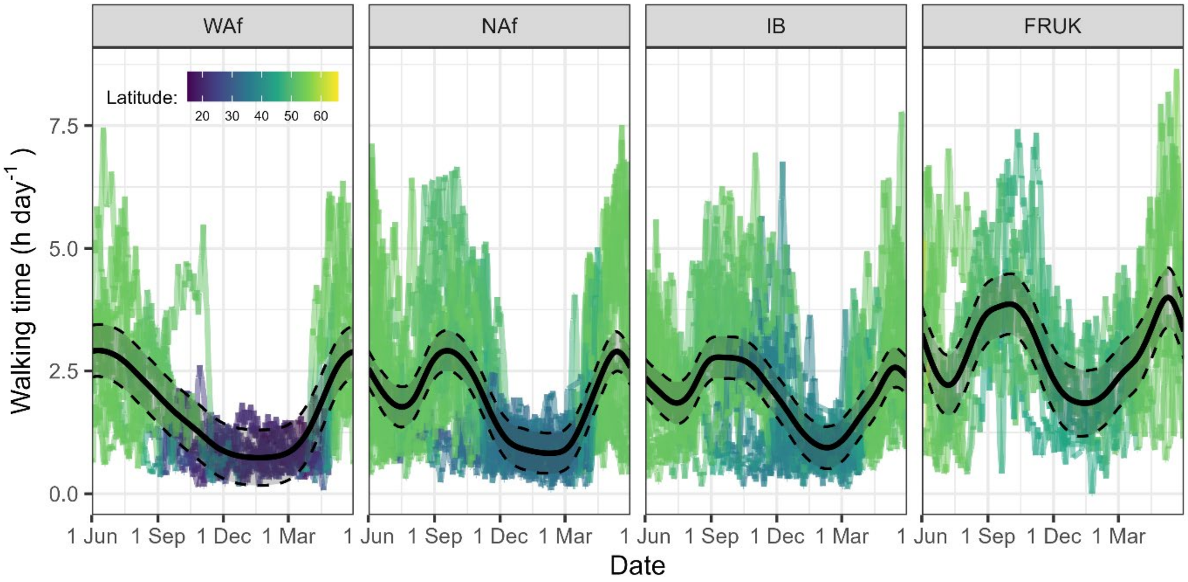

Fig. S3. Daily time spent walking by migration strategy (WAf = West Africa, NAf = North Africa, IB = Iberia, FRUK = France and UK). Bold black line shows GAMM prediction per strategy with approximate 95% confidence intervals. Coloured lines show 7-day mean daily energy expenditure per bird-year, coloured by latitude.

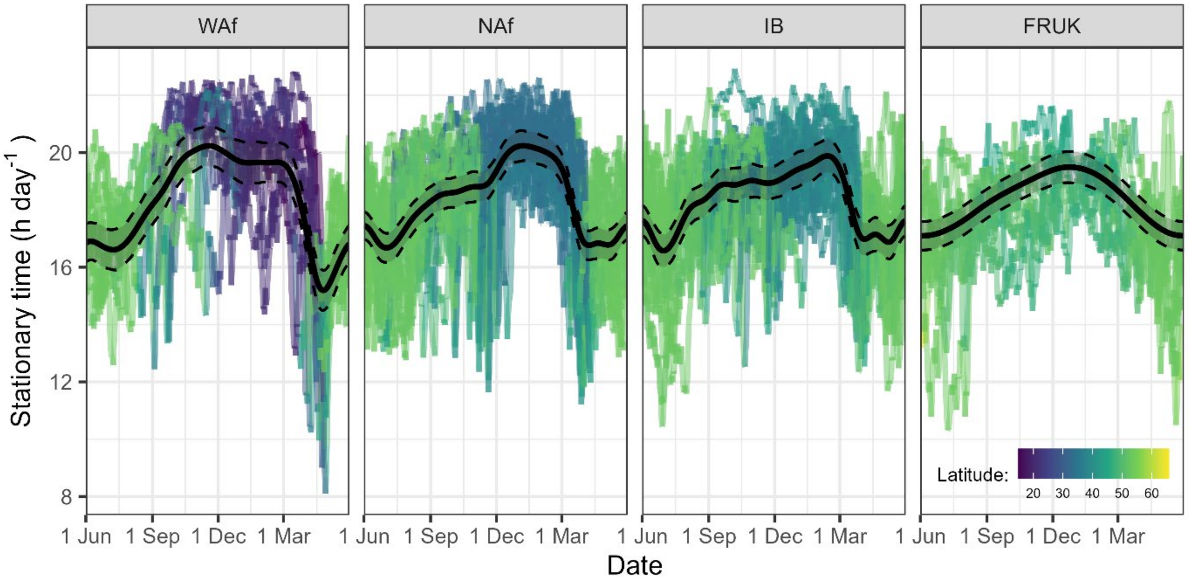

Fig. S4. Daily time spent stationary by migration strategy (WAf = West Africa, NAf = North Africa, IB = Iberia, FRUK = France and UK). Bold black line shows GAMM prediction per strategy with approximate 95% confidence intervals. Coloured lines show 7-day mean daily energy expenditure per bird-year, coloured by latitude.

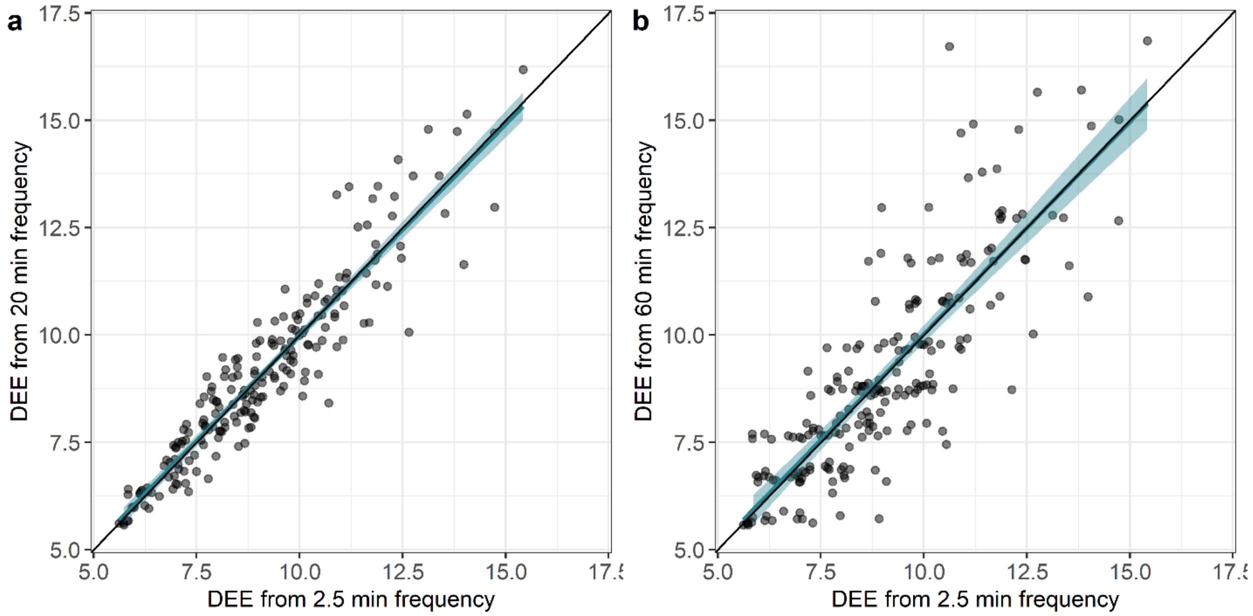

Fig. S5. Comparison of DEE estimated from high resolution (2.5 min) and low resolution (panel a = 20 min, panel b = 60 min) accelerometer data (n = 207 days from 5 lesser black-backed gulls). Black line shows line of equality, and coloured line shows linear relationship between data sets (± standard error).

Table S1.

AIC comparison of GAMM models of daily energy expenditure, flight metabolic rate, time flapping, soaring, in flight (all flight combined), walking, and stationary, flight and stationary metabolic rate, and experienced weather condition, of lesser black-backed gulls throughout their annual cycle. Strat = migration strategy. Models shown in Figs. 4 and 5 are in bold.

. Model	N. Par.	ΔAIC
Daily Energy Expenditure		
f(day)	6	0
f(day) + strat	9	5.4
**f(day*strat)**	9	24.1
f(day*strat) + strat	12	29.5
null	5	186.0
strat	8	190.1
Flight Metabolic Rate		
f(day)	6	0
f(day) + strat	9	0.2
**f(day*strat)**	9	2.6
f(day*strat) + strat	12	2.7
null	5	124.6
strat	8	125.4
Time Flapping		
**f(day)**	6	0
f(day) + strat	9	3.5
f(day*strat)	9	29.4
f(day*strat) + strat	12	32.6
null	5	200.2
strat	8	204.5
Time Soaring		
**f(day)**	6	0
f(day) + strat	9	1.8
f(day*strat)	9	24.3
f(day*strat) + strat	12	26.3
null	5	278.9
strat	8	281.4
Time in Flight		
f(day)	6	0
f(day) + strat	9	3.2
**f(day*strat)**	9	34.4
f(day*strat) + strat	12	37.5
null	5	279.1
strat	8	282.8

. Model	N. Par.	ΔAIC
Time Walking		
f(day) + strat	9	0
**f(day)**	6	12.0
**f(day*strat) + strat**	12	16.2
f(day*strat)	9	29.1
strat	8	200.5
null	5	230.1
Time Stationary		
**f(day)**	6	0
f(day) + strat	9	0.7
**f(day*strat)**	9	19.0
f(day*strat) + strat	12	19.7
strat	5	305.8
null	8	306.7
Stationary Metabolic Rate		
**f(day*strat)**	9	0
f(day*strat) + strat	12	2.0
f(day)	6	85.2
f(day) + strat	9	86.7
strat	8	171.7
null	5	172.1
Temperature		
**f(day*strat) + strat**	12	0
f(day*strat)	9	56.1
f(day) + strat	9	535.8
f(day)	6	586.2
strat	8	1066.5
null	5	1111.9
Solar radiation		
**f(day*strat) + strat**	12	0
f(day*strat)	9	50.9
f(day) + strat	9	364.7
f(day)	6	409.5
strat	8	1041.0
null	5	1052.7

Table S2.

Number of bird-years showing a behavioural pattern suggestive of flight feather moult by annual cycle stage and wintering area.

Winter Area	Breeding	Autumn or winter	No moult pattern
West Africa	3 (30%)	2 (20%)	5 (50%)
North Africa	2 (9%)	13 (59%)	7 (32%)
Iberia	1 (6%)	12 (67%)	5 (28%)
France/UK	0 (0%)	9 (100%)	0 (0%)
